# Cortical control of object‐specific grasp relies on adjustments of both activity and effective connectivity: a common marmoset study

**DOI:** 10.1113/JP274629

**Published:** 2017-09-02

**Authors:** Banty Tia, Mitsuaki Takemi, Akito Kosugi, Elisa Castagnola, Alberto Ansaldo, Takafumi Nakamura, Davide Ricci, Junichi Ushiba, Luciano Fadiga, Atsushi Iriki

**Affiliations:** ^1^ Laboratory for Symbolic Cognitive Development RIKEN Brain Science Institute Saitama Japan; ^2^ Center for Translational Neurophysiology of Speech and Communication Istituto Italiano di Tecnologia Ferrara Italy; ^3^ Graduate School of Science and Technology Keio University Kanagawa Japan; ^4^ Department of Biosciences and Informatics, Faculty of Science and Technology Keio University Kanagawa Japan; ^5^ Keio Institute of Pure and Applied Sciences (KiPAS) Keio University Kanagawa Japan; ^6^ Danish Research Centre for Magnetic Resonance Copenhagen University Hospital Hvidovre Hvidovre Denmark; ^7^ Graphene Labs Istituto Italiano di Tecnologia Genoa Italy

**Keywords:** electrocorticography (ECoG), grip type, marmoset monkey, motor cortex

## Abstract

**Key points:**

- The cortical mechanisms of grasping have been extensively studied in macaques and humans; here, we investigated whether common marmosets could rely on similar mechanisms despite strong differences in hand morphology and grip diversity.
- We recorded electrocorticographic activity over the sensorimotor cortex of two common marmosets during the execution of different grip types, which allowed us to study cortical activity (power spectrum) and physiologically inferred connectivity (phase‐slope index).
- Analyses were performed in beta (16–35 Hz) and gamma (75–100 Hz) frequency bands and our results showed that beta power varied depending on grip type, whereas gamma power displayed clear epoch‐related modulation.
- Strength and direction of inter‐area connectivity varied depending on grip type and epoch.
- These findings suggest that fundamental control mechanisms are conserved across primates and, in future research, marmosets could represent an adequate model to investigate primate brain mechanisms.

**Abstract:**

The cortical mechanisms of grasping have been extensively studied in macaques and humans. Here, we investigated whether common marmosets could rely on similar mechanisms despite striking differences in manual dexterity. Two common marmosets were trained to grasp‐and‐pull three objects eliciting different hand configurations: whole‐hand, finger and scissor grips. The animals were then chronically implanted with 64‐channel electrocorticogram arrays positioned over the left premotor, primary motor and somatosensory cortex. Power spectra, reflecting predominantly cortical activity, and phase‐slope index, reflecting the direction of information flux, were studied in beta (16–35 Hz) and gamma (75–100 Hz) bands. Differences related to grip type, epoch (reach, grasp) and cortical area were statistically assessed. Results showed that whole‐hand and scissor grips triggered stronger beta desynchronization than finger grip. Task epochs clearly modulated gamma power, especially for finger and scissor grips. Considering effective connectivity, finger and scissor grips evoked stronger outflow from primary motor to premotor cortex, whereas whole‐hand grip displayed the opposite pattern. These findings suggest that fundamental control mechanisms, relying on adjustments of cortical activity and connectivity, are conserved across primates. Consistently, marmosets could represent a good model to investigate primate brain mechanisms.

## Key points

- The cortical mechanisms of grasping have been extensively studied in macaques and humans; here, we investigated whether common marmosets could rely on similar mechanisms despite strong differences in hand morphology and grip diversity.
- We recorded electrocorticographic activity over the sensorimotor cortex of two common marmosets during the execution of different grip types, which allowed us to study cortical activity (power spectrum) and physiologically inferred connectivity (phase‐slope index).
- Analyses were performed in beta (16–35 Hz) and gamma (75–100 Hz) frequency bands and our results showed that beta power varied depending on grip type, whereas gamma power displayed clear epoch‐related modulation.
- Strength and direction of inter‐area connectivity varied depending on grip type and epoch.
- These findings suggest that fundamental control mechanisms are conserved across primates and, in future research, marmosets could represent an adequate model to investigate primate brain mechanisms.

**Abbreviations**
AP: antero‐posterior
ECoG: electrocorticography
ERD: event‐related desynchronization
ERS: event‐related synchronization
LFP: local field potential
M1: primary motor cortex
ML: medio‐lateral
PSI: phase‐slope index
SEP: somatosensory‐evoked potential
4‐f, 3a‐f and 3b‐f: forelimb regions of Brodmann areas 4, 3a and 3b, respectively
4‐nf, 3a‐nf, 3b‐nf: non‐forelimb regions of Brodmann areas 4, 3a and 3b, respectively

## Introduction

The basic forelimb structure evolved to allow remarkable feats of manual precision in primates (Mountcastle, 2005; Padberg *et al*. 2007). The cortical control of grasping is founded on the synergistic activity of a parietofrontal network including premotor, primary motor and somatosensory cortex (Fogassi *et al*. 2001; Fluet *et al*. 2010; Gharbawie *et al*. 2011). The premotor cortex modulates corticospinal outputs from primary motor cortex (M1) in a muscle‐ and grasp‐specific manner (Prabhu *et al*. 2009), whereas M1 interacts with the primary somatosensory cortex to integrate proprioceptive and tactile feedback (Salimi *et al*. 1999a,b; Gardner *et al*. 2007).

Although several studies have been conducted in macaques and humans (Salimi *et al*. 1999a,b; Fogassi *et al*. 2001; Gardner *et al*. 2007; Prabhu *et al*. 2009; Fluet *et al*. 2010; Gharbawie *et al*. 2011), evidence remains sparse on the cortical grasping circuits in phylogenetically distant primates, who generally display more rudimentary manual skills. Compared to macaque monkeys, common marmosets present a lower degree of forelimb specialization characterized by an absence of precision grip, reduced object manipulation and little tactile exploration (Coleman *et al*. 2001; Krubitzer & Disbrow, 2008). Marmosets are further characterized by a lissencephalic brain (Newman *et al*. 2009; Kelava *et al*. 2012), and it is generally thought that they lack direct cortico‐motoneuronal projections to distal hand muscles (Lemon & Griffiths, 2005; Kondo *et al*. 2015; Walker *et al*. 2016). The regions corresponding to parietal areas 1 and 2 appear to be merged into one (Krubitzer & Kaas, 1990), displaying low responsiveness to tactile stimuli (Krubitzer & Kaas, 1990; Krubitzer & Disbrow, 2008). Altogether, these features suggest some basic differences in the cortical mechanisms of grasping across species. However, comparative studies indicate that the organization of cortical motor areas and, in particular, cortico‐cortical connections to M1 are rather similar across species (Burman *et al*. 2014; Bakola *et al*. 2015), hinting that some basic mechanism might be conserved.

Albeit several anatomical studies have characterized cortical areas and networks in common marmosets (Krubitzer & Kaas, 1990; Huffman & Krubitzer, 2001; Burish *et al*. 2008; Burman *et al*. 2008, 2014), few investigations were made on their functional properties, and more precisely, in relation to skilled hand movements. The main aim of this study is to shed light on this issue by examining grip adaptation in relation to cortical activity and physiologically inferred connectivity. Specifically, we trained two common marmosets to reach for, grasp and pull three objects, the shapes of which elicited different hand configurations. In parallel, we studied local field potentials (LFPs) obtained from epicortical recording over the sensorimotor cortex. We used event‐related desynchronization/synchronization (ERD/ERS) in beta (16–35 Hz) and gamma (75–100 Hz) bands (Crone *et al*. 1998a,b; Pfurtscheller *et al*. 2003; Miller *et al*. 2007) during the execution of different grip types as the marker of cortical activity. We also calculated the phase‐slope index (PSI) for estimating the effective connectivity between cortical regions (Nolte *et al*. 2008). These allowed us to investigate differences in activity and connectivity related to grip type, task epoch, and cortical area.

Considering that cortical organization and connectivity is highly conserved in primates (Padberg *et al*. 2007), we could hypothesize that marmoset cortex possesses similar cortical finger movement control mechanisms to macaques or humans, despite striking differences in hand morphology (Torigoe, 1985; Coleman *et al*. 2001) and corticospinal connections (Lemon & Griffiths, 2005; Kondo *et al*. 2015; Walker *et al*. 2016). In this case, grip adaptation would rest on adjustments of cortical activity and connectivity among sensorimotor areas (Aoki *et al*. 2001; Davare *et al*. 2008). This would suggest common principles of cerebral cortex function/structure across different species of the primate order (see also Padberg *et al*. 2007). An alternative hypothesis would be that grip adaptation in marmosets relies on processes that are different from cortical mechanisms of more dexterous primates (e.g. subcortical structures; Castiello & Begliomini, 2008).

A secondary aim of this study is to verify whether previously established electrocorticography (ECoG) frequency analysis techniques are applicable to marmosets. Cortical oscillatory activity is generally investigated in alpha (8−13 Hz), beta (15−35 Hz) and gamma (36−100 Hz) frequency bands (Crone *et al*. 1998a,b; Aoki *et al*. 2001; Pfurtscheller *et al*. 2003; Miller *et al*. 2007). These frequencies have been related to movement perception and execution, sensorimotor integration and movement preparation, as well as cognitive processes related to attention, learning and memory formation (Başar *et al*. 2001; Engel & Fries, 2010; Cheyne, 2013). In particular, beta (Spinks *et al*. 2008; Turella *et al*. 2016) and gamma power (Aoki *et al*. 1999, 2001; Miller *et al*. 2007) are modulated by manual tasks. Beta LFP is known to exhibit amplitude decrease during movement in relatively broad sensorimotor areas, whereas gamma LFP displays power increase during movement in relevant focal brain areas in both macaques and humans (Crone *et al*. 1998a,b; Pfurtscheller *et al*. 2003; Miller *et al*. 2007). However, movement‐related modulation within these frequency bands has not yet been documented in common marmosets.

Finally, this investigation allowed us to assess the usefulness of micro‐ECoG arrays purpose‐built in our laboratory. These electrodes were coated with nanomaterials (Castagnola *et al*. 2013, 2014), reducing impedance and increasing charge injection capacity, thus making it possible to use them for both recording and stimulation. Moreover, because of the thin dura of lissencephalic marmoset brain (Bourne & Rosa, 2003; Lui *et al*. 2014), the array was placed epidurally, which dramatically reduces damage to cortical tissue, and should guarantee stability of LFP signals over long‐term implantation (Yeager *et al*. 2008; Slutzky *et al*. 2011; Komatsu *et al*. 2015).

## Methods

### Ethical approval

The data presented here were recorded from two purpose‐bred adult marmoset monkeys (*Callithrix jacchus*: MK1, male, 2.8 years, 305 g; MK2, male, 4.6 years, 352 g), housed at the RIKEN Brain Science Institute (Wako, Japan). The animals were not food deprived, but in order to maintain a high motivation for the task, daily food was provided at the end of each testing session. Water was always available *ad libitum*. All procedures were performed in accordance with the Laboratory Animal Welfare Act and *The Guide for the Care and Use of Laboratory Animals* (National Institutes of Health, Bethesda, MD, USA) and were approved by the Institutional Animal Research Committee at RIKEN (IRB approval number H24‐2‐228). All adequate measures were taken to minimize pain or discomfort.

### Task

**Figure 1:**
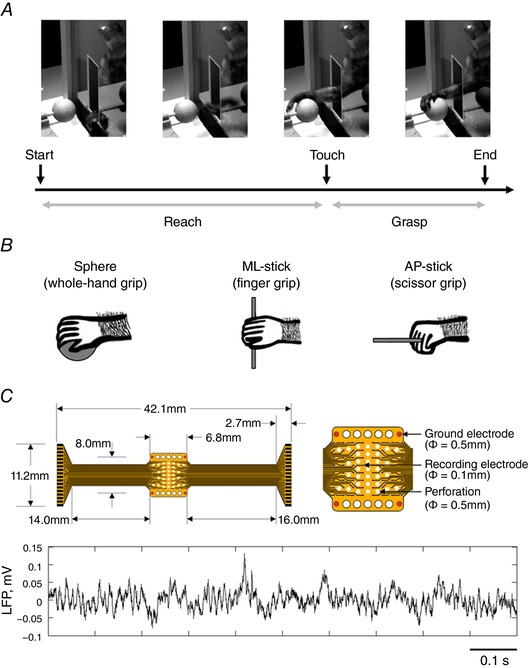
Experimental set‐up *A*, image sequence of the behavioural task. Two epochs were identified: ‘reach’ (from onset of hand movement until touch) and ‘grasp’ (from touch until end of pulling action). *B*, objects presented and hand postures used by the monkey to grasp them. The animals were trained to reach for, grasp and pull a sphere (whole‐hand grip), a stick oriented along the medio‐lateral axis of the animal (ML‐stick; finger grip) and a stick oriented along the antero‐posterior axis of the animal (AP‐stick; scissor grip). *C*, LFPs were recorded using 63‐channel ECoG arrays. The upper panels display an entire array (left) and a zoom‐in of the recording area (right). The lower panel displays LFP activity recorded from a single channel during a representative trial.

Recording sessions were conducted with the monkey free to move in an experimental cage (surface, 28.5 cm × 28.5 cm; height, 29.0 cm). The cage included a transparent acrylic door with an opening (width, 3.0 cm; height, 7.0 cm; distance from the floor, 6.0 cm) that allowed the monkey to reach for, grasp and pull an object located at a distance of 3.0 cm (Fig. 1 *A*). The object had to be pulled by 1.0 cm from the starting point. After reaching the target position, the monkey released the object and received a food reward from the experimenter. The task was performed with the arm contralateral to the recording side. This was ensured by configuring the apparatus so that the target was only reachable by the contralateral arm. The training phase took about 2 months. Monkeys typically performed a total of 10–30 successful grasping actions during a standard recording session. Hand movements were monitored throughout the experiments by a video camera at 30 frames s^−1^ (HDR‐HC9, Sony Corporation, Japan).

### Objects used

The objects presented to the monkeys were selected to evoke different grip types according to their intrinsic physical characteristics (Fig. 1 *B*). The sphere (diameter, 25 mm) elicited a whole‐hand grip involving the palm and fingers. The stick oriented medio‐laterally with respect to the animal (ML‐stick; diameter, 2 mm; length, 75 mm) solicited more extensively the fingers and scarcely involved the palm. The stick oriented antero‐posteriorly with respect to the animal (AP‐stick; diameter, 2 mm; length, 75 mm) elicited a scissor grip that consisted of pinching the object between two adjacent fingers.

### Electrocorticogram (ECoG) arrays

LFPs were recorded over the sensorimotor cortex using 8 × 8 micro‐ECoG arrays (Fig. 1 *C*) purpose‐built in our laboratory. Contact diameter was 100 μm and inter‐electrode distance was 900 μm in the medio‐lateral (ML) and 700 μm in the antero‐posterior (AP) direction. Electrodes were coated with a nanocomposite of poly‐(3,4 ethylene‐dioxythiophene) and carbon nanotubes, and encapsulated in fibrin hydrogel (Castagnola *et al*. 2013, 2014). The use of nanomaterial coatings reduced electrode impedance and increased charge injection capacity, making it possible to use the electrode for both recording and stimulation. In addition, the presence of multiple perforations through the device ensured an effective contact with the brain surface and the free flow of cerebrospinal fluid.

### Surgery

Anaesthesia was induced with intraperitoneal injection of medetomidine, midazolam and butorphanol (0.05 mg kg^−1^, 0.5 mg kg^−1^ and 0.5 mg kg^−1^, respectively). Atropine (0.10 mg kg^−1^) and prednisolone (0.15 mg kg^−1^) were intramuscularly injected immediately after the anaesthesia. During the surgery, anaesthesia was maintained by inhalation of 1.5–2.5% isoflurane and the oxygen saturation level was continuously monitored. When intensive post‐surgical care was required, the animals were anaesthetized with isoflurane (0.5–3%) and administrated with lidocaine (subcutaneous injection) for analgesia.

For implantation of a sheet of ECoG electrode array, a craniotomy of 9 × 5 mm (approx. coordinates relative to bregma: 0–9 mm anterior and 2–7 mm lateral) was performed in the left hemisphere and the dura remained intact. The ECoG sheet was implanted between the dura and the skull. In non‐human primates, the ECoG sheet is usually implanted subdurally due to the thickness of the dura mater which is likely to reduce signal accuracy (Moran, 2010). However, in marmosets, the dura is relatively thin compared to macaques and humans (Bourne & Rosa, 2003; Lui *et al*. 2014), allowing high quality recording through epidural implant (Komatsu *et al*. 2015), similarly to experimental protocols in rats (Yeager *et al*. 2008; Slutzky *et al*. 2011). The sheet was laid onto the dura using a micromanipulator, and then a piece of artificial dura mater was placed between the array and the skull in order to maintain the electrode in place. The piece of artificial dura was cut longer and narrower than the electrode sheet, so that the anterior and posterior edges of the artificial dura could be placed under the skull. Since the medial and lateral sides of the electrode sheet were also under the skull, this procedure allowed the electrode to be maintained in the correct position. The artificial dura also helped to avoid the dura getting dry, and thus it could keep a better signal‐to‐noise ratio. A head chamber made of Ultem (height, 15 mm; width, 18 mm; length, 16 mm), a polyetherimide polymer with high dielectric strength, solvent resistance and mechanical properties, was attached to the skull with stainless screws and dental acrylic. The purpose of using the chamber was to reduce risks of infection by isolating the tissues within the chamber. Furthermore, in order to improve stability of the recording, the inside of the chamber was filled with silicone adhesive (Kwik‐Cast, World Precision Instruments, Sarasota, FL, USA) one week after surgery. This time lapse was necessary to stabilize brain pressure, which is generally considered unstable just after surgery. Both brain swelling and shrinking might have led to, respectively, under‐ and over‐estimate the amount of silicone adhesive.

### Recording of local field potentials

The electrode array (63 recording channels) was connected to a wireless headstage (Triangle Biosystems International, Durham, NC, USA), and the signals were filtered (1–1000 Hz) and digitized at 2713 Hz using the Digital Lynx data acquisition system (Neuralynx, Bozeman, MT, USA). Four electrodes (0.5 mm in the diameter) located at each corner of the ECoG array acted as a linked reference.

### Recording locations

**Figure 2:**
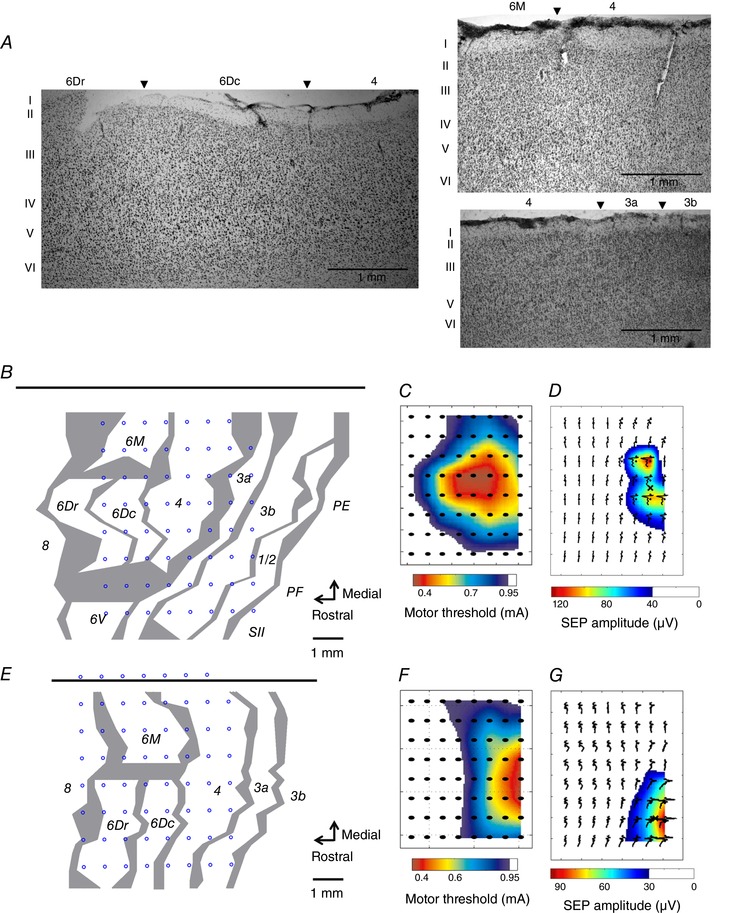
ECoG array location over the sensorimotor cortex *A*, parasagittal sections illustrating the cytoarchitectural characteristics of premotor (6Dr, 6Dc, 6M), primary motor (4) and somatosensory (3a, 3b) fields. *B* and *E*, reconstruction of array location over the cortex by histological assessment for MK1 (*B*) and MK2 (*E*). The top black horizontal line represents the midline. Grey lines indicate architectonic borders, reconstructed from sagittal sections. The thickness of the lines represents zones of uncertainty. Blue circles refer to the location of each of the 63 recording electrodes. *C* and *F*, primary motor forelimb representation for MK1 (*C*) and MK2 (*F*). Motor maps were composed of motor thresholds that are intensities of epicortical stimulation eliciting electromyographic activity of forelimb muscle (extensor digitorum communis). *D* and *G*, somatosensory forelimb representation for MK1 (*D*) and MK2 (*G*). Somatosensory maps were composed of somatosensory‐evoked potential (SEP) amplitudes elicited by median nerve stimulation.

In order to identify the cortical regions covered by the array, histological mapping was performed at the end of the experiment (Fig. 2 *A*, *B* and *E*). Additionally, forelimb motor representation (Fig. 2 *C* and *F*) and somatosensory representation (Fig. 2 *D* and G) were estimated in the course of the experiment.

#### Histological map

At the end of the experimental study, the monkey was anaesthetized with ketamine (15 mg kg^−1^) and pentobarbital (75 mg kg^−1^). The animal was perfused through the heart with phosphate‐buffered saline and paraformaldehyde. The postmortem brain was measured and photographed. A block of cortex containing the recorded areas was cut parasagittally and 50 μm histological sections were mounted and Nissl stained. Histological assessment allowed screening for cytoarchitectonic evidence of tissue damage, if any, associated with the use of epidural ECoG arrays (Molina‐Luna *et al*. 2007). Next, the recording location of the entire array over the cortex was reconstructed (Fig. 2 *B* and *E*). Histological borders were plotted as transition zones of various width, reflecting sources of uncertainty such as test–retest variability (assessed by repeated plotting by the same observer on different days) and interference of histology artifacts. Criteria described in previous studies (Krubitzer & Kaas, 1990; Burman *et al*. 2006, 2008, 2015) were used to distinguish between premotor, primary motor and somatosensory areas. Briefly, Brodmann area 4, or M1, is characterized by the absence of layer IV and the presence of large pyramidal cells in layer V (Fig. 2 *A*). Rostrally, area 6Dc of the premotor cortex is distinguished by similar features to area 4, with smaller cells in layer V. Area 6Dr has properties intermediate between area 6Dc and granular areas (8b, 8aD and 8aV), comprising the presence of a less defined layer IV compared to prefrontal areas, and the absence of large pyramidal cells in layer V. Area 6M is medial to area 6Dc and comprises an incipient layer IV, as opposed to areas 4 and 6Dc, and large cells in layer V. Area 6V, lateral to area 6Dc, is characterized by a thin layer IV, large pyramidal neurons at the base of layer III and large cells in layer V. Caudally to area 4, area 3a of the somatosensory cortex comprises an identifiable layer IV and large cells in layer V, although smaller than those present in area 4. Area 3b has well‐defined layers IV and VI, and a thin layer V with small pyramidal cells. Area 1/2 is characterized by less densely packed layers IV and VI than 3b.

#### Forelimb motor map

Forelimb motor maps (Fig. 2 *C* and *F*) were obtained as follows. First, approximately 1 week before the ECoG implantation surgery, two multistranded stainless steel wires (AS634, Cooner Wire, Chatsworth, CA, USA), spaced 5 mm apart, were chronically implanted in the right extensor digitorum communis muscle that extends the four medial digits of the hand. During surgery, the animals were anaesthetized following the same protocol as previously described for ECoG array implantation. Electrical cortical stimulation was applied through the ECoG electrodes while the animals were awake. The ECoG channel used for stimulation was randomly selected at each trial. The stimulus train consisted of 5 biphasic pulses (250 μs cathodal and 250 μs anodal) delivered at 1000 Hz, and the maximum stimulator output was adjusted to 1.0 mA. Stimulus current was generated by an isolated output source (SS‐203J; Nihon Kohden, Tokyo, Japan) and controlled from an analog output module (NI PCIe‐6321; National Instruments). Electromyographic signals were recorded, band‐pass filtered (1–2000 Hz), and digitized at 4800 Hz using a biosignal amplifier (g.USBamp; g.tec medical engineering GmbH, Graz, Austria). The motor threshold, an intensity eliciting muscle twitch with 50% of probability, was estimated for each channel using the maximum likelihood method (Awiszus, 2003). We repeated cortical stimulation mapping four times within one day, and the topographic profile of the motor thresholds across channels, so‐called forelimb motor map, was defined. We then divided the histologically defined area 4 into the forelimb region (4‐f) and the non‐forelimb region (4‐nf).

#### Forelimb somatosensory map

The forelimb somatosensory map (Fig. 2 *D* and G) was estimated on the basis of somatosensory‐evoked potentials (SEPs) obtained by stimulation of the median nerve contralateral to the recorded hemisphere. SEPs were induced by transcutaneous electric stimulation applied via two electrodes (diameter, 1 mm) placed over the median nerve of awake animals. Stimuli consisting of square wave impulses of 2 ms with current intensity between 0.3 and 0.5 mA were applied 200 times at a frequency of 2 Hz. SEP response size was then calculated for each channel in order to obtain a topographic profile of the forelimb somatosensory representation. Histologically defined areas 3a and 3b were divided into the forelimb regions (3a‐f, 3b‐f) and the non‐forelimb regions (3a‐nf, 3b‐nf) based on the result of forelimb somatosensory map.

### Data analysis

**Figure 3:**
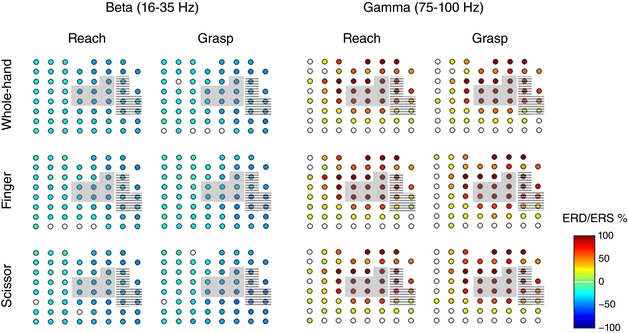
Event‐related desynchronization (ERD) in beta band and event‐related synchronization (ERS) in gamma band over the sensorimotor cortex of MK1 Differences from the baseline resting power were statistically assessed by the bootstrap method (*P* < 0.05). Statistically significant ERD/ERSs were represented as filled coloured circles, whereas the electrodes with non‐significant ERD/ERSs were represented as open circles. Blue denotes ERD and red denotes ERS, ranging from −100% to 100%, respectively (scale presented in the lower right corner). Bad channels were excluded from analysis and were not represented. The grey and striped zones correspond to primary motor and somatosensory forelimb representations, respectively.

**Figure 4:**
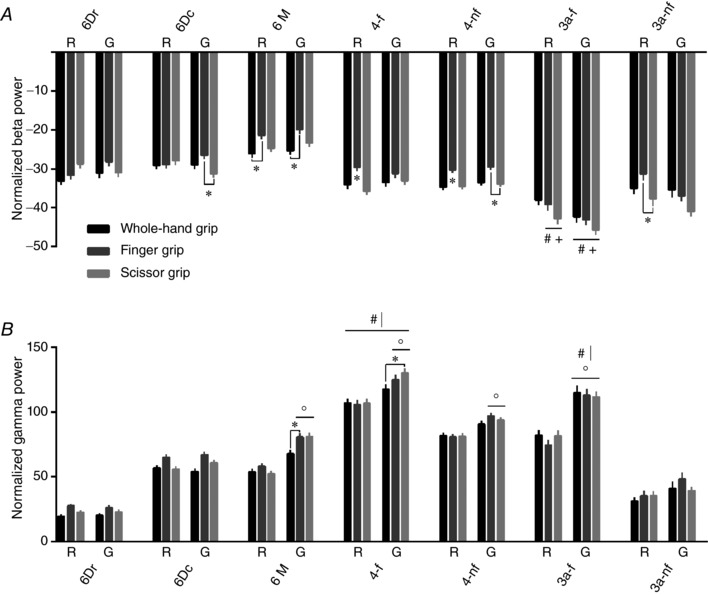
Area, epoch and grip‐related normalized power in beta (*A*) and gamma (*B*) bands Power value for each channel was normalized with respect to the baseline resting power. Channels were then grouped according to cortical area, and data from both animals were pooled in a single ensemble. Pairwise comparisons were performed using a Wilcoxon rank‐sum test with a Bonferroni‐Holm correction, following three‐way ANOVA with aligned rank transform (factors: cortical area, task‐related epoch, grip type). All measurements are expressed as mean ± SEM. ^*^ *P* < 0.05, different from other grip type(s); °*P* < 0.05, different from other epoch; ^#^ *P* < 0.05, different from premotor areas 6Dr, 6Dc and 6M; ^+^ *P* < 0.05, different from primary motor areas 4‐f and 4‐nf; **^|^** *P* < 0.05, different from somatosensory area 3a‐nf. R, reach; G, grasp. 6Dr, 6Dc, 6M, 4 and 3a refer to the corresponding Brodmann areas. ‐f, forelimb representation; ‐nf, non‐forelimb representation.

Analyses were performed only on data recorded during successful trials, namely when the monkey completed the reach, grasp and pull action without necessary repositioning of the hand on the object. Combining successful trials resulted in a pooled ensemble of ≈ 85 trials per grip type for each monkey. Two task‐related epochs were defined (Fig. 1 *A*): ‘reach’, from onset of hand movement until object touch, and ‘grasp’, from touch until end of pulling action. LFP data were cut around reach and grasp epochs. The segments including large amplitude artifacts, which are problematic for accurate independent component analysis (ICA) decomposition, as well as saturated channels, were removed (Rogasch *et al*. 2014). ICA was then applied to the concatenated data to remove components reflecting residual artifacts. In particular, independent components of which the weighted matrix showed spatial discontinuity due to sudden amplitude fluctuations in one channel (Mognon *et al*. 2011) were subtracted from the concatenated signals. In order to study the activity evoked at each site for the different grip types and epochs, the artifacts‐free data that were segmented into each epoch were analysed using fast Fourier transform. Power spectra averaged across beta (16–35 Hz) and gamma (75–100 Hz) bands were then estimated, because beta LFP is known to exhibit amplitude decrease during movement in a relatively broad sensorimotor area and gamma LFP represents a spectrally broad power increase that accompanies movement in relevant focal brain areas (Crone *et al*. 1998a,b; Pfurtscheller *et al*. 2003; Miller *et al*. 2007). In the present study, ERD/ERS showed changes in power spectrum relative to the resting period, expressed as percentages of the resting power. Statistical significance of ERD/ERS was assessed by the bootstrap method (*P* < 0.05; Fig. 3; Graimann *et al*. 2002). Finally, to identify differences between conditions, we performed three‐way ANOVA with aligned rank transform, with factors of ‘grip’ (whole‐hand grip, finger grip, scissor grip), ‘epoch’ (reach, grasp) and ‘area’ (6Dr, 6Dc, 6M, 4‐f, 4‐nf, 3a‐f, 3a‐nf) followed by *post hoc* pairwise comparisons using a Wilcoxon rank‐sum test with a Bonferroni‐Holm correction (*P* < 0.05; Fig. 4).

**Figure 5:**
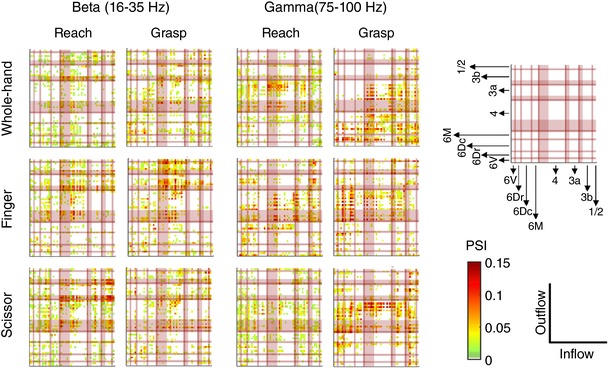
PSI values obtained in beta and gamma bands for MK1 Each coloured point represents the outflow from channel ‘i’ designated by the *y*‐coordinate, to channel ‘j’ designated by the *x*‐coordinate. Statistically significant values were represented as coloured points, whereas non‐significant values were left white. For each pair of channels, PSI was retained as significant only if greater than 95% of values computed on shuffled data (1000 shuffling times), thus corresponding to a one‐tailed probability value of *P* < 0.05. In each plot, lines delimit the channels located within different cortical areas. 6V, 6Dr, 6Dc, 6M, 4, 3a, 3b and 1/2 refer to the corresponding Brodmann areas. Shaded zones of area 4, 3a and 3b correspond to the forelimb representation.

**Figure 6:**
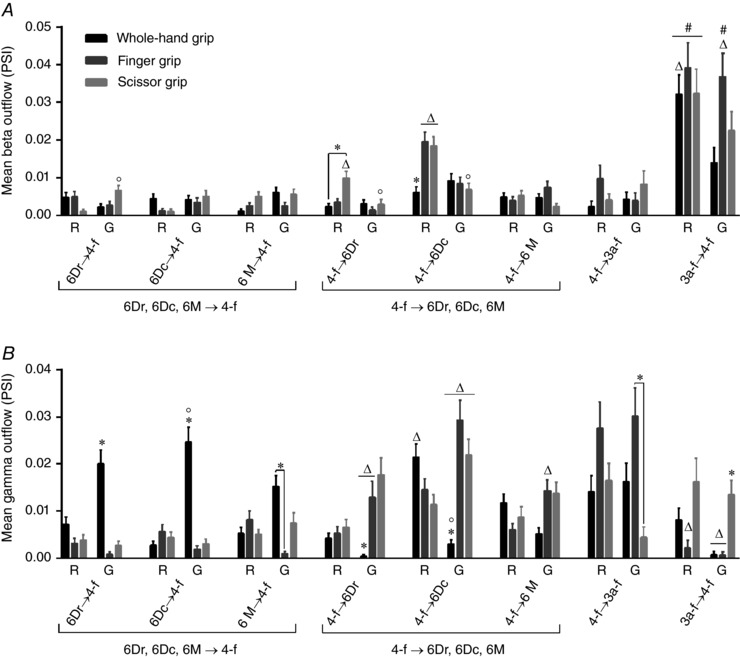
Epoch and grip‐related outflow among the recorded areas, in beta (*A*) and gamma (*B*) bands Pairwise comparisons were performed using a Wilcoxon rank‐sum test with a Bonferroni‐Holm correction, following three‐way ANOVA with aligned rank transform (factors: direction of outflow, task‐related epoch, grip type). All measurements are expressed as mean ± SEM. ^*^ *P* < 0.05, different from other grip type(s); °*P* < 0.05, different from other epoch; ^Δ^ *P* < 0.05, different from outflow in opposite direction; ^#^ *P* < 0.05, different from outflow from premotor areas 6Dr, 6Dc, 6M to primary motor cortex 4‐f. R, reach; G, grasp. 6Dr, 6Dc, 6M, 4 and 3a refer to the corresponding Brodmann areas. ‐f, forelimb representation; ‐nf, non‐forelimb representation.

In order to study the direction of information flow between sensorimotor areas, we computed PSI which gives a measure of the outflow/inflow resulting from interactions between sites. PSI analysis is based on the slope of the phase of the cross‐spectrum between two sites. It was shown to be insensitive to mixtures of independent sources, to yield meaningful results even with non‐linear phase spectra, and to properly weight contributions from different frequencies (Nolte *et al*. 2008). To perform this analysis, all artifact‐free data relative to a given grip type and epoch were truncated to keep only the central 250 ms‐bin. Trials were concatenated and PSI values in the beta and gamma bands were calculated for all channel pairs. PSI was tested for significance by a permutation procedure that involved creating 1000 permuted versions of the LFP dataset, in which trial order was independently permuted for each site (Fig. 5; Edgington, 1980; Brovelli *et al*. 2004). This procedure had the effect of disrupting task‐related PSI and yielding PSI values due to chance. For each pair of channels, PSI was retained as significant only if greater than 95% of values computed on shuffled data, thus corresponding to a one‐tailed probability value of *P* < 0.05. Next, to assess differences between conditions, we performed three‐way ANOVA with aligned rank transform, with factors of ‘grip’ (whole‐hand grip, finger grip, scissor grip), ‘epoch’ (reach, grasp) and ‘direction’ (6Dr to 4‐f, 4‐f to 6Dr, 6Dc to 4‐f, 4‐f to 6Dc, 6M to 4‐f, 4‐f to 6M, 4‐f to 3a‐f, 3a‐f to 4‐f) followed by *post hoc* pairwise comparisons using a Wilcoxon rank‐sum test with a Bonferroni‐Holm correction (*P* < 0.05; Fig. 6).

## Results

### Database

This study is based on a total of 514 recordings (i.e. grasping trials) made during 39 experimental sessions in two hemispheres of two common marmosets, MK1 and MK2. In MK1, a total of 266 successful recordings were obtained in 27 experimental sessions over a period of 5 weeks. In MK2, a total of 248 successful recordings were obtained in 12 experimental sessions over a period of 3 weeks. For each monkey and grip type, the 25th, 50th and 75th percentiles of reach and grasp duration are presented in Table 1. Additional recordings were made to measure baseline cortical activity in MK1 (450 recordings) and MK2 (463 recordings).

**Table 1:** Duration of reach and grasp epochs for whole‐hand, finger and scissor grips, in MK1 and MK2

	Action	Grip	25th percentile (ms)	50th percentile (ms)	75th percentile (ms)
MK1	Reach	Whole‐hand	367	467	633
		Finger	300	467	692
		Scissor	400	500	800
	Grasp	Whole‐hand	333	433	600
		Finger	300	400	567
		Scissor	400	517	633
MK2	Reach	Whole‐hand	333	400	500
		Finger	300	400	533
		Scissor	300	400	467
	Grasp	Whole‐hand	433	533	600
		Finger	333	467	567
		Scissor	500	567	633

### Recording sites

The histological examination confirmed minimal damage of the implanted areas, as revealed by their cytoarchitectonic features (Fig. 2 *A*). Together with the absence of motor deficit in the behavioural task, this observation demonstrates the safety of the experimental procedure (see also Molina‐Luna *et al*. 2007). The histological assessment further revealed that, in MK1, recordings were mainly made from premotor (area 6V, 6Dr, 6Dc, 6M), M1 (area 4) and somatosensory (area 3a, 3b, 1/2) cortex in the left hemisphere (Fig. 2 *B*). In MK2, recordings were made from prefrontal (area 8), premotor (area 6Dr, 6Dc, 6M), primary motor (area 4) and somatosensory (area 3a) cortex in the left hemisphere (Fig. 2 *E*). For both monkeys, a subset of electrodes was located over the forelimb representation of M1 (MK1, 7; MK2, 6; Fig. 2 *C* and *F*) and somatosensory (MK1, 6; MK2, 2; Fig. 2 *D* and *G*) cortex. Regions of area 4, 3a and 3b corresponding to the forelimb representation are respectively designated as 4‐f, 3a‐f and 3b‐f.

### Modulation of beta and gamma power during the task

In order to assess task‐related power modulations in the sensorimotor cortex, ERD/ERS was calculated for each channel in the beta (16–35 Hz) and gamma (75–100 Hz) bands. For both animals, beta ERD and gamma ERS were observed over most recorded areas, for all three grip types, during the reach and grasp epochs (MK1, Fig. 3). This finding confirmed the reliability of the recorded signal which was consistent with well‐known movement‐related power modulations (Crone *et al*. 1998a,b; Pfurtscheller *et al*. 2003; Miller *et al*. 2007).

Three‐way ANOVA with aligned rank transform was performed on data from both animals and was pooled in a single ensemble. These analyses yielded several significant effects and pairwise differences, as described below.

#### Beta band (Fig. 4 *A*)

The ANOVA performed on normalized beta power revealed significant main effects of grip (*F*_2,44515_ = 55.5, *P* < 0.001), epoch (*F*_1,44515_ = 17.0, *P* < 0.001) and area (*F*_6,44515_ = 148.4, *P* < 0.001). In addition, significant interactions grip × area (*F*_12,44515_ = 2.5, *P* < 0.01), epoch × area (*F*_6,44515_ = 2.9, *P* < 0.01) and grip × epoch × area (*F*_12,44515_ = 2.1, *P* < 0.05) were detected. When comparing grip types, beta ERD was on average stronger for scissor grip (‐32.1 ± 0.3%) and whole‐hand grip (−32.1 ± 0.3%) compared to finger grip (−29.0 ± 0.3%). Consistently, a number of *post hoc* comparisons showed stronger ERD for scissor grip (area 6Dc, grasp epoch; area 4‐f, reach epoch; area 4‐nf, reach and grasp epochs; area 3a‐nf, reach epoch) and whole‐hand grip (area 6M, reach and grasp epochs; area 4‐f, reach epoch; area 4‐nf, reach epoch) compared to finger grip (*P* < 0.05). This finding is consistent with previous research in macaques showing grip‐related differences in beta power during stable hold (Spinks *et al*. 2008).

When comparing cortical areas, somatosensory area 3a‐f showed stronger ERD than premotor and primary motor areas (finger and scissor grips, reach epoch; whole‐hand, finger and scissor grips, grasp epoch; *P* < 0.05). Except for this effect, beta ERD was similar across sensorimotor regions, in agreement with the literature indicating a diffuse distribution over cortical regions (Szurhaj & Derambure, 2006).

#### Gamma band (Fig. 4 *B*)

The ANOVA performed on normalized gamma power revealed significant main effects of ‘grip’ (*F*_2,44515_ = 11.0, *P* < 0.001), ‘epoch’ (*F*_1,44515_ = 217.2, *P* < 0.001) and ‘area’ (*F*_6,44515_ = 755.9, *P* < 0.001). In addition, significant interactions grip × epoch (*F*_2,44515_ = 13.9, *P* < 0.001) and epoch × area (*F*_6,44515_ = 28.3, *P* < 0.001) were detected. Normalized gamma ERS was on average highest for finger grip (72.7 ± 0.9%), intermediate for scissor grip (70.4 ± 0.8%) and lowest for whole‐hand grip (67.7 ± 0.8%). However, *post hoc* comparisons showed few grip‐related differences, which consisted of stronger ERS for finger grip (area 6M, grasp epoch) and scissor grip (area 4‐f, grasp epoch) compared to whole‐hand grip (*P* < 0.05). By contrast, several epoch differences were detected, with stronger gamma ERS during grasp than reach epoch (area 6M, area 4‐f, area 4‐nf: finger and scissor grips; area 3a‐f: whole‐hand, finger and scissor grips; *P* < 0.05). It is important to note that this pattern, which reflects an adjustment in cortical activity throughout the task, was more consistently observed for finger and scissor grips than whole‐hand grip. Overall, these results confirm the idea that gamma oscillations encode movement parameters (Schalk *et al*. 2007; Mollazadeh *et al*. 2011).

Finally, some area differences emerged, as illustrated by stronger ERS for area 4‐f (reach and grasp epochs) and 3a‐f (grasp epoch) than 6Dr, 6Dc, 6M and 3a‐nf (*P* < 0.05). In agreement with previous work (Szurhaj & Derambure, 2006; Miller *et al*. 2007), our results suggest that strong gamma ERS was somewhat spatially specific and concentrated in primary sensorimotor forelimb representation.

### Direction and strength of outflow depend on grip type and epoch

In order to study the effective connectivity between cortical areas, PSI was computed for all channel pairs. Figure 5 illustrates the connectivity patterns obtained for MK1 in the different experimental conditions, in beta and gamma bands. Only significant values, as determined by the permutation method (one‐tailed, *P* < 0.05), were displayed. Information flow occurred among all recorded areas and appeared to vary depending on grip type and epoch. Different patterns seemed to arise in beta and gamma bands, in agreement with the idea that these two frequency ranges cover different functions.

To statistically determine whether connectivity differed across grip types, epochs and directions of outflow, we performed three‐way ANOVA with aligned rank transform, followed by pairwise comparisons using a Wilcoxon rank‐sum test with a Bonferroni‐Holm correction (Fig. 6). For this analysis, the data from both animals were pooled in a single ensemble. Significant pairwise differences are described below.

#### Beta band (Fig. 6 *A*)

The ANOVA performed on beta outflow revealed significant main effects of ‘grip’ (*F*_2,3648_ = 69.4, *P* < 0.001), ‘epoch’ (*F*_1,3648_ = 195.9, *P* < 0.001) and ‘direction’ (*F*_7,3648_ = 80.3, *P* < 0.001). Moreover, significant interactions grip × epoch (*F*_2,3648_ = 26.9, *P* < 0.001), grip × direction (*F*_14,3648_ = 16.6, *P* < 0.001), epoch × direction (*F*_7,3648_ = 44.6, *P* < 0.001) and grip × epoch × direction (*F*_14,3648_ = 18.6, *P* < 0.001) were found.

Pairwise comparisons showed minor grip‐related differences. Namely, finger grip triggered stronger outflow than whole‐hand grip (from 4‐f to 6Dc; reach epoch). Scissor grip also triggered stronger outflow than whole‐hand grip (from 4‐f to 6Dr and 6Dc; reach epoch; *P* < 0.05). These results suggest that strong beta outflow from M1 to premotor cortex is crucial to hand preshaping for small objects (finger and scissor grips), requiring fine adjustments of finger kinematics and forces.

Furthermore, regarding scissor grip, epoch differences emerged that consisted of stronger outflow during reach than grasp (from 4‐f to 6Dr and 6Dc) and stronger outflow during grasp than reach (from 6Dr to 4‐f; *P* < 0.05). This variation shows that, contrary to other grip types, scissor grip requires significant connectivity adjustments in beta band during the task.

Finally, comparisons between directions of outflow revealed that, for finger and scissor grips, during reach, outflow from area 4‐f to 6Dc outweighed the opposite pathway (*P* < 0.05). For scissor grip, this was also verified when considering outflow from 4‐f to 6Dr. These findings confirm the previous observation that hand preshaping for small objects requires a strong contribution of M1.

Altogether, our findings are consistent with evidence in other species showing a modulation of premotor–M1 interactions during different types of grasp (see Prabhu *et al*. 2009 for data for macaque; Davare *et al*. 2008 for human data). However, to our knowledge, this is the first study to report changes in the dominant direction of inter‐area connectivity, depending on grip type and task epoch.

Regarding somatosensory–M1 connectivity, outflow from 3a‐f to 4‐f was found to be stronger than the opposite pathway, for whole‐hand grip (reach epoch) and finger grip (grasp epochs), whereas it did not reach significance for scissor grip. Besides, for all grip types during reach, outflow from somatosensory area 3a‐f exceeded outflow from premotor areas 6Dr, 6Dc and 6M to primary motor area 4‐f. For finger grip, this pattern was also verified during grasp. Overall, these results are consistent with the crucial role of tactile/proprioceptive information during grasping (see also Brovelli *et al*. 2004 for data for macaques; Filimon, 2010; Babiloni *et al*. 2016 for human data). Our results further confirm previous experiments in macaques showing strong directed coherence from primary somatosensory cortex to M1 in beta band (Witham *et al*. 2010).

#### Gamma band (Fig. 6 *B*)

The ANOVA performed on gamma outflow revealed significant main effects of ‘grip’ (*F*_2,3648_ = 21.6, *P* < 0.001), ‘epoch’ (*F*_1,3648_ = 85.9, *P* < 0.001) and ‘direction’ (*F*_7,3648_ = 19.7, *P* < 0.001). Also, significant interactions grip × direction (*F*_14,3648_ = 8.2, *P* < 0.001), epoch × direction (*F*_7,3648_ = 5.7, *P* < 0.001) and grip × epoch × direction (*F*_14,3648_ = 14.7, *P* < 0.001) were found.

Pairwise comparisons showed remarkable grip‐related differences in premotor–primary motor connectivity, in particular during the grasp epoch. Whole‐hand grip triggered stronger outflow than finger grip (from 6Dr, 6Dc and 6M to 4‐f; *P* < 0.05) and scissor grip (from 6Dr and 6Dc to 4‐f). Conversely, finger and scissor grips elicited stronger outflow than whole‐hand grip (from 4‐f to 6Dr and 6Dc). These results, in agreement with those obtained in beta band, and consistent with previous work in the literature (Davare *et al*. 2008; Prabhu *et al*. 2009), indicate that the gamma oscillatory network contains clearly distinct premotor–primary motor networks associated with whole‐hand grip *vs*. finger and scissor grips.

Minor epoch differences emerged for whole‐hand grip only, that consisted of stronger outflow during grasp than reach (from 6Dc to 4‐f) and stronger outflow during reach than grasp (from 4‐f to 6Dc).

Finally, comparisons between directions of outflow unveiled some distinctions, especially during grasp, that generally confirmed the previously described effects. In particular, for whole‐hand grip, outflow from premotor areas 6Dr and 6Dc to primary motor area 4‐f exceeded outflow in the opposite direction (*P* < 0.05). By contrast, for finger and scissor grips, outflow from 4‐f to 6Dc outweighed the opposite pathway. For finger grip, this was also verified from 4‐f to 6Dr and 4‐f to 6M.

Altogether, these results confirm those obtained in beta band, albeit with more significant grip‐related differences. In other words, in gamma frequencies, the dominant direction of inter‐area connectivity appeared to vary depending on grip type. Grasping small objects (finger and scissor grips) was associated with stronger outflow from primary motor to premotor cortex. To our knowledge, this is the first study to report these results in common marmosets.

Besides premotor–primary motor interactions, additional findings emerged when examining primary motor–somatosensory connectivity. Outflow from 4‐f to 3a‐f exceeded outflow from 3a‐f to 4‐f, for whole‐hand grip during grasp epoch and for finger grip during reach and grasp epochs. Further, finger grip induced stronger outflow than scissor grip, from 4‐f to 3a‐f during grasp epoch. Conversely, scissor grip elicited stronger outflow than finger and whole‐hand grips, from 3a‐f to 4‐f during grasp epoch. These results suggest that whole‐hand and finger grips rest on different primary motor–somatosensory interactions compared to the scissor grip, when considering gamma oscillations.

## Discussion

In this experimental study, we examined the epicortical activity in sensorimotor cortices of two common marmosets performing whole‐hand, finger and scissor grips. Reach (from onset of hand movement until touch) and grasp (from touch until end of pull) epochs were distinguished in order to identify possible adaptation of the cortical signal throughout the task. The aim of this investigation was to identify patterns of cortical activity and effective connectivity characterizing different grip types in common marmosets. Our results showed that beta power was clearly modulated by grip type, whereas gamma power was more distinctly modulated by task epoch, reflecting adjustments in cortical activity during the task, in particular for finger and scissor grips. Moreover, different strengths of outflow/inflow between cortical regions were detected depending on grip type and epoch. The implications of these results are discussed below.

### Common marmosets display classical movement‐related power modulations

We found ERD in beta band (16–35 Hz) and ERS in gamma band (75–100 Hz) for most recording sites over the sensorimotor cortex of both monkeys. Beta ERD was rather evenly distributed over premotor, primary motor and somatosensory cortex, albeit somatosensory area 3a‐f showed stronger ERD than motor areas. Gamma oscillations displayed stronger ERS over primary motor and somatosensory forelimb representations, compared to premotor areas. These distributions in beta and gamma bands appear consistent with previous work in macaques (Sanes & Donoghue, 1993; Kilavik *et al*. 2013) and humans (Crone *et al*. 1998a,b; Aoki *et al*. 2001; Pfurtscheller *et al*. 2003; Miller *et al*. 2007; Darvas *et al*. 2010) and confirm the reliability of the present experimental procedure.

### Local synchrony between neural populations is task dependent

In the literature, low and high frequency task‐related changes in spectral power have been attributed to alterations in synchrony between neural populations around recording sites (Aoki *et al*. 1999; Pfurtscheller *et al*. 2003; Canolty *et al*. 2006; Miller *et al*. 2009). Our results demonstrated that beta and gamma power over sensorimotor cortex respectively displayed substantial grip‐ and epoch‐related modulations. In particular, stronger beta ERD was detected for scissor and whole‐hand grips compared to finger grip, in several premotor and primary motor regions. With regard to gamma band, stronger ERS was found during grasp than reach epoch in several sensorimotor areas. These epoch differences were more consistently observed for finger and scissor grips – associated with small objects – than whole‐hand grip.

There is evidence that substantial information on grasping features, including kinematics and forces, is contained in LFPs (Pistohl *et al*. 2012; Milekovic *et al*. 2015). However, intermediate frequencies (10–45 Hz) are known to provide smaller classification and decoding accuracy than low (<7 Hz) and high (75–250 Hz) frequencies (Schalk *et al*. 2007; Mollazadeh *et al*. 2008, 2011; Kubanek *et al*. 2009; Pistohl *et al*. 2012; Milekovic *et al*. 2015). In fact, the issue of beta power modulation by movement parameters is still controversial (Kilavik *et al*. 2013). In humans, no difference was found between precision and side grips in terms of beta ERD (Zaepffel *et al*. 2013) although, by contrast, reaching and grasping tasks induced distinct, action‐specific, modulation (Turella *et al*. 2016). Previous lack of grip‐specific effects might in part be due to experimental protocols including complex hand configurations associated with independent finger control, and excluding more simple hand postures such as hook or power grips (Zaepffel *et al*. 2013). Indeed, in macaques, Spinks *et al*. (2008) demonstrated that complex grips involving the thumb (e.g. precision grip) were associated with lower beta power, whereas simpler grips involving flexion of the four medial fingers (e.g. hook grip) were associated with higher beta power, as obtained from intracortical LFPs in ventral premotor and primary motor cortex. This selectivity was essentially evidenced during static hold rather than actual movement (Spinks *et al*. 2008). During static postural maintenance, beta rhythm in the motor cortex synchronizes with motor unit activity, and may partly drive the tonic muscle contraction, although conversely, muscle reafference could drive cortical activity (Baker *et al*. 1997; Kilavik *et al*. 2013). In the present study, we showed that marmosets display grip selectivity of beta ERD in premotor cortex and M1. Direct comparison with a previous work in macaques (Spinks *et al*. 2008) is rendered difficult by the difference in tested grips, due to differences in the anatomy and function of the thumb among species (Napier, 1961; Torigoe, 1985; Krubitzer & Disbrow, 2008). Notably, we detected this grip‐related variation in beta ERD during actual forelimb movement, as opposed to static hold in macaques (Spinks *et al*. 2008), suggesting that additional mechanisms might be recruited.

Considering gamma oscillations, previous investigation in humans described power modulation during different hand motor control tasks such as target tracking, threading and sequential pinches (Aoki *et al*. 1999, 2001; Miller *et al*. 2007). In the present work, minor differences arose related to grip type in marmosets. However, we showed a number of differences between epochs in sensorimotor cortex, for a subset of grips – namely finger and scissor grips – associated with small objects. To our knowledge, no identical results have been reported in macaques or humans, although it is well‐known that gamma oscillations encode movement kinematics (Schalk *et al*. 2007; Mollazadeh *et al*. 2008, 2011; Kubanek *et al*. 2009; Pistohl *et al*. 2012; Milekovic *et al*. 2015) that varies across task epochs (Roy *et al*. 2000; Castiello, 2005; Takemi *et al*. 2014). Our results are compatible with single‐unit studies pointing out that populations of neurons show different patterns of activity depending on grip type and task epoch (Umiltà *et al*. 2007). In fact, epoch‐related variations in gamma power might reflect a mechanism for movement adaptation during more challenging tasks, which require precise monitoring of hand and digit movements, in common marmosets.

### Strength of connectivity between cortical areas differs across grip types and epochs

In order to better understand the interactions between cortical regions, we estimated the direction of information flow by using the PSI (Nolte *et al*. 2008). Our results showed that whole‐hand grip relies on stronger interaction from premotor cortex to M1, whereas finger and scissor grips rely on stronger interaction from M1 to premotor cortex. These results were mainly present in gamma band during the grasp epoch. Moreover, minor differences between epochs were detected, in beta band for scissor grip and gamma band for whole‐hand grip. This result illustrated a small degree of adjustment in premotor–primary motor connectivity throughout the prehensile task.

These modulations of premotor–primary motor connectivity in common marmosets are in agreement with previous findings in macaques (Prabhu *et al*. 2009) and humans (Davare *et al*. 2008, 2009), which attest that cortical interactions are selectively adjusted during specific types of grasp. Earlier studies demonstrated that, both in macaques and humans, premotor cortex can modulate corticospinal outputs through M1 in a muscle‐ and grasp‐specific manner (Davare *et al*. 2009; Prabhu *et al*. 2009). In humans, transcranial magnetic stimulation experiments further showed that ventral premotor cortex exerts a net inhibitory influence on M1 at rest, whereas this inhibition disappears during power grip, and is converted into a net facilitation during precision grip (Davare *et al*. 2008).

What could explain PSI differences depending on grip type in marmosets? We should consider that PSI is a requisite of causality from one region to the other, but not sufficient evidence of the direct influence. This means a causal influence from M1 to premotor cortex via intermediate regions, such as cerebellar nuclei, is shown as a significant PSI from M1 to premotor cortex. By taking this limitation of PSI into consideration, the grip‐related differences in connectivity evidenced here can be interpreted in terms of feedforward and feedback controllers (Shadmehr & Krakauer, 2008; Scott, 2012). Literature from the field of computational neuroscience suggests that M1, known as a feedback controller, sends a copy of motor command to cerebellum, known as the feedforward controller. The feedforward controller uses the copy to predict the sensory consequences of the command. In order to adjust the current motor command generated in M1, this prediction is compared with the sensory feedback resulting from actual movements in the dorsal premotor and parietal cortex suggested as state estimators. Here, we showed that for finger and scissor grips, information flux, resulting from bidirectional premotor–primary motor interactions, was predominantly directed from M1 to premotor cortex. This result could potentially illustrate a major role of the motor command copies and the sensory prediction to optimize the movement when grasping small objects (Wolpert, 1997; Chersi *et al*. 2011; Hill *et al*. 2011). Besides, this result enhances the crucial role of M1 for fine adjustment of kinematics and forces directed to small objects, as has largely been documented (Kakei *et al*. 1999; Saleh *et al*. 2010; Pistohl *et al*. 2012).

Finally, considering primary motor–somatosensory interactions, future investigations, with more detailed study of somatosensory forelimb representation, would be desirable to strengthen the validity of our findings. One noticeable result consisted of major beta outflow from somatosensory to motor cortex, and major gamma outflow from motor to somatosensory cortex. This finding, in line with previous investigations, suggests that a beta oscillatory network is involved in the transfer of sensory signals from somatosensory cortex to M1 (see also, in macaques, Brochier *et al*. 1999; Brovelli *et al*. 2004; Gardner *et al*. 2007; Witham *et al*. 2010), whereas gamma oscillations could intervene in the prediction of sensory consequences of actions (Desmurget & Grafton, 2000; Shadmehr *et al*. 2010).

### Beta and gamma oscillations might represent different physiological phenomena

In agreement with the literature in other primates, our findings support the notion that, in common marmosets, beta and gamma oscillatory networks cover different physiological phenomena (Miller *et al*. 2007). Analysis of beta ERD showed a number of grip‐related differences, whereas the majority of differences in gamma ERS were epoch related, albeit these were more consistently observed only in a subset of grips. With regard to premotor–primary motor connectivity, grip‐related differences were mostly detected in gamma band during the grasp epoch (i.e. from the object touch until the end of pull). These distinctions between the two frequency bands are in line with a modelling work that suggests that beta and gamma oscillations may play different functional roles in neural communication and processing (Kopell *et al*. 2000). Indeed, low frequency changes were postulated to originate in broad cortical areas, collectively regulated by central structures (e.g. thalamus and basal ganglia; Cassidy *et al*. 2002; Paradiso *et al*. 2004; Foffani *et al*. 2005; Miller *et al*. 2007). In these areas, distributed populations of neurons are active to different extents for different types of grasp, resulting in different overall discharge (Umiltà *et al*. 2007) and LFP power (Spinks *et al*. 2008). By contrast, high frequency changes were suggested to reflect the integrated activity of local neuronal populations immediately underneath the electrodes (Hoogenboom *et al*. 2006; Womelsdorf *et al*. 2006; Miller *et al*. 2007). Therefore, we can speculate that high frequencies could more accurately mediate interactions between localized neuronal populations, resulting in more substantial task‐specific connectivity.

### Usefulness of our experimental protocol for future investigations

The final aspects to highlight from this study are its methodological advantages. The surgical procedure was minimally invasive. The ECoG sheets were implanted epidurally with minimal damage to the brain tissue. Further, the arrays, purpose‐built in our laboratory (Castagnola *et al*. 2013, 2014), allowed both recording and stimulation over an extended time period (chronic implant; recording period: MK1, 5 weeks, MK2, 3 weeks). Applying this minimally invasive technology to the marmoset, which has lissencephalic brain and thin dura and yet presents typical primate characteristics (Rosa & Tweedale, 2005; Newman *et al*. 2009; Kelava *et al*. 2012; Burman *et al*. 2014; Bakola *et al*. 2015), could provide an efficient future application for the research field, particularly in terms of long‐term experimentation such as learning/developmental plasticity, ageing‐related changes, and mental disorders (Johnson *et al*. 1996; Pryce *et al*. 2004, 2011). Note that, although unit activities directly measured from neurons provide temporally and spatially precise information, both high‐gamma and low‐beta oscillations in LFPs are known to be correlated with neural firing rate (Ray *et al*. 2008; Spinks *et al*. 2008), suggesting that LFPs are fairly well able to illustrate neuronal activities in the brain.

In addition, marmosets present several advantages as a primate model. They are especially easy to raise and handle in laboratory conditions due to their small body size, and are characterized by fast sexual maturation (Lu *et al*. 2001; Okano *et al*. 2012; Mitchell *et al*. 2014). Also, the ease of use of marmosets for molecular genetic studies (Sequencing & Consortium, 2014) would further expand the possibilities of the research field, for instance, to investigate the molecular basis of specific neural circuitries involved in certain behaviours (e.g. Iriki *et al*. 1996; Ishibashi *et al*. 2002; Hihara *et al*. 2006; Quallo *et al*. 2009).

Finally, from a phylogenetic perspective, by comparing cortical mechanisms associated with different hand morphologies of marmosets, macaques and humans, our protocol could provide opportunities to investigate the evolutionary significance of hand control in primate strains (Lui & Rosa, 2014).

### Concluding remarks

Our results support the idea that, in common marmosets, grip adaptation correlates to adjustments of cortical activity and physiologically inferred connectivity. Therefore, despite striking differences in manual dexterity, marmosets seemingly rely on similar mechanisms to those previously identified in macaques and humans. This conservation of fundamental control principles across primates suggests that marmosets could represent a good model to investigate primate brain mechanisms. However, species differences in prehensile abilities, as illustrated by the lack of an opposable thumb and reduced grip diversity in marmosets (Napier, 1961; Torigoe, 1985; Krubitzer & Disbrow, 2008), could have led researchers to exclude the possibility of similar mechanisms. Since direct cross‐species comparisons, with identical techniques and protocols, are rare (Peeters *et al*. 2009; Mars *et al*. 2011), neurophysiological interpretations of species differences should be taken with caution. One possibility is that more dexterous primates are characterized by stronger grip‐related selectivity than less dexterous ones, when considering other signal components than those tested here, for example single‐unit firing rates (Murata *et al*. 1997; Raos *et al*. 2006; Spinks *et al*. 2008) or decoding of LFP signal (Schalk *et al*. 2007; Zhuang *et al*. 2010; Flint *et al*. 2012; Pistohl *et al*. 2012). Moreover, in macaques and humans, evidence suggests that grip type modulates cortical interactions in terms of strength of connectivity (Davare *et al*. 2008, 2009) or sites within a given area (Murata *et al*. 2000; Umiltà *et al*. 2007; Bonini *et al*. 2012), whereas there are no distinct reports of changes in the direction of inter‐area information flux. Further research is necessary to better characterize the cortical grasping circuits that evolved along with prehensile specializations in primates. In future studies, more elaborate tasks in terms of motor/cognitive complexity would allow more in‐depth examination of cortical functions, such as the contribution of premotor cortex and M1 with regard to goal‐directedness.

## Additional information

### Competing interests

A.I. is the president and CEO of Rikaenalysis Corporation (RIKEN Venture, Tokyo). The other authors declare no competing financial interests.

### Author contributions

This study was conducted in the laboratory of A.I. at RIKEN Brain Science Institute, Wako, Japan. B.T., M.T., J.U., L.F. and A.I. conceived and designed the study. M.T., E.C., A.A. and D.R. conceived experimental devices. B.T., M.T., A.K. and T.N. performed data collection. B.T., M.T. and A.K. analysed the data. B.T., M.T., A.K., J.U., L.F. and A.I. drafted the manuscript. E.C., A.A., T.N. and D.R. provided critical revisions. All authors have approved the final version of the manuscript and agree to be accountable for all aspects of the work. All persons designated as authors qualify for authorship, and all those who qualify for authorship are listed.

### Funding

This work was supported by a grant provided for the Brain/MINDS project to A.I. (by AMED, Japan), Grants‐in‐Aid for JSPS Research Fellow to M.T. (no. 14J00630) and by grants from the Italian Ministry of the University and Research to L.F. (PRIN) in collaboration with the Robotics Brain and Cognitive Sciences Department of the Italian Institute of Technology.
